# What Causes the Cough in Whooping Cough?

**DOI:** 10.1128/mbio.00917-22

**Published:** 2022-05-23

**Authors:** Nicholas Carbonetti

**Affiliations:** a University of Maryland School of Medicine, Baltimore, Maryland, USA

**Keywords:** *Bordetella pertussis*, cough, mouse model, pertussis, whooping cough

## Abstract

What causes the cough in whooping cough (pertussis) has been a longstanding question in the field but has been difficult to answer because of the perceived lack of convenient small animal models. Y. Hiramatsu, K. Suzuki, T. Nishida, N. Onoda, et al. (mBio 13:e01397-21, 2022, https://doi.org/10.1128/mbio.03197-21) used a mouse model and cellular studies to investigate bacterial and host factors that contribute to cough production during Bordetella pertussis infection. In elegant studies, they found that the bacterial factors pertussis toxin, lipooligosaccharide, and Vag8 function cooperatively to produce cough. These factors induce production of host bradykinin, a known cough inducer that sensitizes the ion channel TRPV1 on neurons, and they investigated host signaling pathways altered by the bacterial factors that exacerbate cough responses. This is a highly significant and important finding that not only elucidates mechanisms underlying the pathophysiology of the severe cough, but also may reveal potential novel therapeutic approaches to treat individuals suffering from the debilitating effects of cough in pertussis.

## COMMENTARY

Whooping cough, or pertussis, is notable for the severity of the cough. Individuals suffering from pertussis are typically unable to stop coughing once a bout has started, and the severity and longevity of these paroxysmal coughing bouts is painful and debilitating. To make matters worse, there are no effective therapeutics to treat this condition. But what causes pertussis cough, and why is the cough so severe? Some in the field have speculated on the production of a “cough toxin” by Bordetella pertussis, the bacterial agent that causes pertussis. The feasibility of this idea was reinforced by the recent finding that a single glycolipid component of Mycobacterium tuberculosis, sulfolipid-1, can induce cough in experimentally infected guinea pigs (1). However, no such B. pertussis factor has been identified to date. Part of the problem was that mouse models were long considered inappropriate to investigate this question—many (including cough experts!) believed that mice cannot cough, even though they can be experimentally infected by B. pertussis. Recent reports have changed this view, demonstrating that mice do cough in response to certain stimuli, and that the cough can be detected by sensitive recording equipment (2, 3).

In the report by Hiramatsu et al. (4), the authors used mouse models to demonstrate that experimental B. pertussis infection causes cough and to investigate the bacterial and host factors that contribute to this response. Mice started coughing about a week into the infection and continued to cough for at least another week after that. Bacterial lysates could also induce cough, indicating that specific components were responsible rather than infection by live bacteria. They showed that one of these components is pertussis toxin (PTx), a secreted protein toxin that inhibits signaling through G_i_-linked G protein-coupled receptors (GPCRs) in mammalian cells. This was consistent with a previous report showing that rats experimentally infected with a wild-type B. pertussis strain, but not an isogenic mutant strain deficient in PTx production, coughed in response to infection (5). However, PTx alone was not sufficient to induce cough, and they identified two additional B. pertussis factors, Vag8 and lipooligosaccharide (LOS), that in combination with PTx could induce cough in mice to the same extent as the bacterial lysate. Vag8 is a surface autotransporter protein that inhibits host complement C1 inhibitor protein (C1-Inh) and contributes to bacterial resistance to complement, and LOS is the major outer membrane component of B. pertussis that signals through the host pattern recognition receptor TLR4 (Toll-like receptor 4) to induce inflammatory responses. The enigmatic “cough toxin” of B. pertussis turns out to be a combination of these three factors!

They then addressed host factors that contribute to this cough response. Using a series of inhibitors of transient receptor potential (TRP) ion channels and GPCRs (since these factors are known to participate in some pathways of cough induction), they identified the bradykinin (Bdk) B2 receptor and the TRPV1 channel as important contributors to the cough response to B. pertussis lysate or the three components PTx, Vag8, and LOS. The role of Bdk in cough induction is well established, and at least one article previously speculated on the role of Bdk in pertussis cough (6). The authors show that Bdk levels in the airways rise during B. pertussis infection or treatment with the three components, but this increase (and the number of coughs) is attenuated in TLR4 knockout mice, indicating a mechanism by which LOS may contribute to cough. They also show that Vag8 enhances Bdk levels and that this activity is due to its binding to (and presumably inhibition of) C1-Inh, an inhibitor of Bdk production. A similar finding on this role of Vag8 in activating the contact system that results in the generation of Bdk was reported previously by Hovingh et al. (7). The involvement of Bdk in cough responses is complex, however, since it signals through both G_q_-linked GPCRs to induce protein kinase C (PKC) activity, which activates TRPV1, and G_i_-linked GPCRs, which can downregulate responses.

The role of TRPV1 in pertussis cough responses, which the authors confirmed with knockout mice, is novel and exciting, although a link between TRP channels and other cough responses is established (8). Using patch clamp and intracellular calcium assay techniques after stimulation of cells with capsaicin (a ligand for TRPV1), the authors addressed the role of PTx in the cough responses. They showed that Bdk inhibits desensitization of TRPV1 activation by a second capsaicin treatment and that PTx further exacerbates this effect, amplifying responses to capsaicin. This effect of PTx was mediated through protein kinase A (PKA), a cAMP-dependent kinase (PTx increases cAMP levels in cells) that phosphorylates and activates TRPV1. They then addressed another possible mechanism by which PTx exacerbates cough responses, involving the α_2_-adrenergic receptor, signaling through which can reduce cough (and pain) responses. They showed that noradrenaline, a ligand for this receptor, inhibits capsaicin-induced TRPV1 activity and the Bdk-induced sensitization of TRPV1, but that this inhibitory activity is abolished by PTx treatment. These effects of PTx may explain the inability of individuals with pertussis to stop coughing once a bout of cough has started, giving the characteristic paroxysmal nature to the cough. Two inherent mechanisms to suppress cough responses once started—that is Bdk signaling through G_i_-linked B2 receptors and noradrenaline signaling through the G_i_-linked α_2_-adrenergic receptor—are both blocked by PTx. The authors do not specifically address the paroxysmal nature of the cough in their infected mice, but a plethysmography trace of airflow in a coughing mouse shows at least one series of several coughs in quick succession.

The authors also begin to address a couple of questions that arose from their study. One question was why the B. pertussis strain 18-323, phylogenetically an outlier among B. pertussis strains, was able to induce cough, while the commonly used strain Tohama was unable to do so. They found that, even though 18-323 produced higher levels of LOS than Tohama, the Bdk levels induced by the two strains or by the three purified components from each were equivalent. However, Tohama lysate was not able to induce coughing, from which they speculate that Tohama produces an unknown inhibitor of cough production. This is at odds with the previous rat studies, in which Tohama was able to induce coughing (5). However, Tohama could not induce coughing in the more recently developed baboon model of pertussis, whereas a recent clinical B. pertussis isolate caused full-blown pertussis disease in these animals (9). Another puzzling observation from the study was that while C57BL/6 mice coughed in response to B. pertussis infection, BALB/c mice did not. The authors showed that BALB/c mice produce lower levels of Bdk in response to infection than C57BL/6 mice and mention other studies that show that BALB/c mice have lower levels of TRPV1 expression in some sets of sensory neurons. Along with known genetic differences between the two mouse strains in their inflammatory responses to infection, these may provide possible reasons for the lack of cough in BALB/c mice.

The latter point brings up the question of how relevant this mouse model is to human pertussis disease. Although mice represent a powerful research tool for discovery purposes, there are many differences between mice and humans, and potentially exciting findings in mouse models do not always translate well to humans. Whether the cough produced by mice, as studied in this report, is physiologically and neurologically similar to that in humans remains to be determined. If there are substantial similarities, then this model should open the doorway to both greater understanding of the basis of pertussis cough and identify potential therapeutic targets for treatment. Fortunately, the pertussis field now has a nonhuman primate model, baboons, which suffer full-blown disease symptoms after experimental infection with B. pertussis. The baboon model is likely more representative of human disease pathophysiology, and therefore, selected findings revealed in the mouse model can be tested in baboons to confirm their involvement in cough and to assess therapeutic potential for humans.

The current study reveals some candidate therapeutic targets from the analysis of host molecules and signaling pathways involved in the production and modulation of cough in response to B. pertussis infection. A leading candidate is the TRPV1 ion channel, identified in the current study as important for the generation of pertussis cough and previously linked with cough responses to other stimuli (10). TRPV1 antagonists have been tested in human trials for effects on cough of noninfectious origin (11). Protein kinase C and PKA, kinases that phosphorylate TRPV1 to stimulate its activity, are other potential targets, although their involvement in a multitude of cellular signaling pathways increases the chances of side effects. Targeting these molecules would presumably overcome the effects of PTx, since each is downstream from PTx modification of G proteins. This long-lived effect of PTx on cells is a likely contributor to the extended period of time over which individuals with pertussis suffer from the severe bouts of paroxysmal coughing. One thing is for sure—any novel and effective therapeutics would be welcome indeed by these individuals!
